# Lung metastasis with primary conjunctival mucoepidermoid carcinoma

**DOI:** 10.22336/rjo.2025.89

**Published:** 2025

**Authors:** Francisco Calleja-Casado, Amparo Lanuza-García, Gemma Ortega-Prades, Sergio Alfredo Maugard-Tepper, Antonio Duch Samper

**Affiliations:** 1 Ophthalmology Department, Hospital Clínico Universitario de Valencia, Valencia, Spain; 2 University of Valencia, Valencia, Spain

**Keywords:** conjunctival squamous carcinoma, conjunctival epidermoid carcinoma, conjunctival mucoepidermoid carcinoma, lung metastases, conjunctival lesion, p63, BAC = Bronchial Aspiration Cytology, BCVA = Best Corrected Visual Acuity, CK = Cytokeratin, CT = Computed Tomography, DM2 = Diabetes Mellitus Type 2, HCV = Hepatitis C Virus, HE = Hematoxylin-Eosin, HIV = Human Immunodeficiency Virus, HPV = Human Papillomavirus, IOP = Intraocular Pressure, MALT = Mucosa-Associated Lymphoid Tissue, MRI = Magnetic Resonance Imaging, OD = Oculus Dexter (Right Eye), OS = Oculus Sinister (Left Eye), OSSN = Ocular Surface Squamous Neoplasia, PET-CT = Positron Emission Tomography-Computed Tomography

## Abstract

**Background:**

Mucoepidermoid carcinoma of the conjunctiva is a rare, aggressive variant of squamous cell carcinoma, with limited reports of systemic metastasis.

**Case presentation:**

An 86-year-old man presented with a suspicious conjunctival lesion. Initial biopsy was inconclusive, but excision confirmed moderately differentiated mucoepidermoid carcinoma. Despite treatment, the tumor showed rapid orbital invasion. Imaging later revealed bilateral lung and liver metastases. Cytology confirmed metastatic spread. The patient died two months later.

**Discussion:**

Mucoepidermoid carcinoma of the conjunctiva is an uncommon and particularly aggressive variant of ocular surface squamous neoplasia, often underdiagnosed due to its mixed histological components. Compared with classic squamous cell carcinoma, it demonstrates higher rates of local recurrence, deeper orbital invasion, and a greater potential for regional spread. Although distant metastasis is exceedingly rare, our case illustrates that this subtype may disseminate rapidly and extensively despite early intervention. These findings highlight the importance of performing deep biopsies to avoid sampling error, ensuring complete histopathological assessment, and considering early systemic imaging when tumor behavior appears unusually aggressive.

**Conclusion:**

Conjunctival mucoepidermoid carcinoma may exhibit aggressive local and systemic behavior. Early deep biopsy, complete histological analysis, and systemic imaging are essential to guide appropriate management and detect rare but fatal metastases.

## Introduction

Squamous or epidermoid carcinoma of the conjunctiva is at the end of a spectrum of ocular surface lesions called ocular surface squamous neoplasia (OSSN) [**1**]. It predominantly affects older people, and significant risk factors include UV light exposure, smoking, human immunodeficiency virus (HIV), and human papillomavirus (HPV16) [**1-3**].

In clinical settings, it typically presents as a tumor on the ocular surface, causing irritative symptoms such as redness and foreign body sensation, though it is often painless at first. Its growth is generally slow but progressive. It usually localizes to the interpalpebral fissure, more commonly on the nasal side [**1,2,4**].

Treatment starts with surgical excision of the lesion, with margins of 2-4 mm if the lesion is small and confined to the conjunctiva; however, the recurrence rate is relatively high, depending on the pathologic analysis [**1,5-8**]. Adjuvant therapies such as local cryotherapy, brachytherapy, or local chemotherapy have traditionally been used to reduce recurrences. If the lesion is larger or invades other orbital structures, aggressive techniques such as orbital exenteration are employed [**1,2,9**].

If treated early, metastasis is extremely rare. If it occurs, it most commonly affects locoregional lymph nodes or adjacent intra- or extraorbital structures. Distant metastases have been reported in isolated cases, generally at advanced stages at diagnosis [**1,2,5-7,10-13**].

## Case report

We present the case of an 86-year-old patient who was first admitted to our hospital’s emergency department for evaluation of a conjunctival lesion in the right eye (OD) that appeared inflamed and caused discomfort, foreign body sensation, and stinging.

Systemic history: Hepatic cirrhosis due to HCV, HTN, DM2.

Oncological history: Gastric Mucosa-Associated Lymphoid Tissue (MALT) lymphoma diagnosed 4 years earlier, with a recurrence after 2 years, non-invasive papillary urothelial carcinoma 33 years before, with two recurrences after 20 and 25 years, with no further recurrences. The patient had no family history of malignancy.

**Fig. 1:**
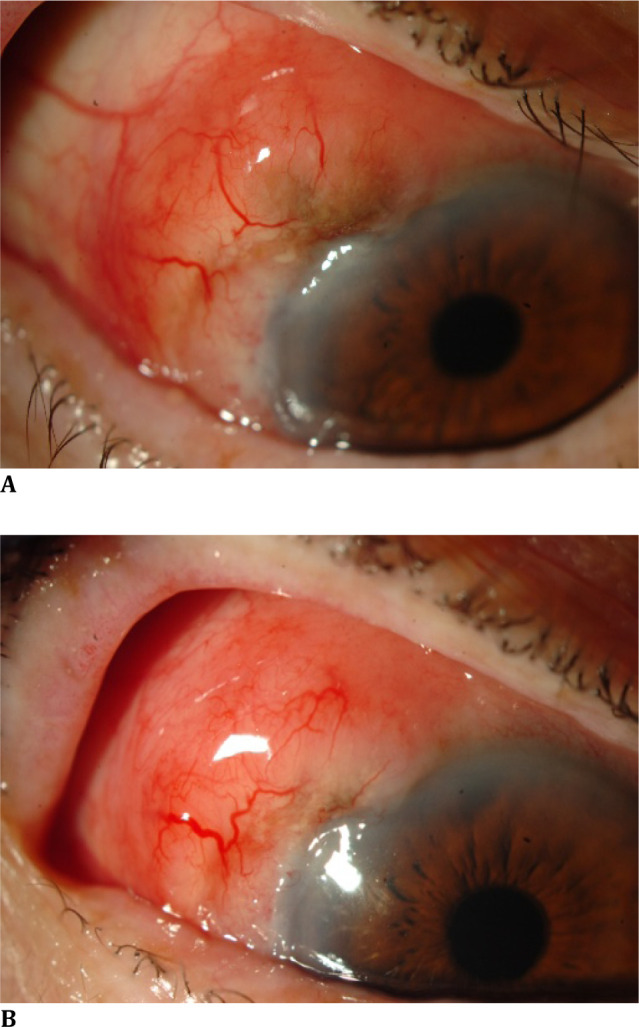
**A, B** Suspicious conjunctival lesion in the OD

Abdominal ultrasound and abdominopelvic computed tomography (CT) were performed that same week for an inguinal hernia, revealing normal hepatic structure without nodules and normal lung bases, except for multiple pleural calcifications compatible with asbestos exposure. Ophthalmologic examination showed best corrected visual acuity (BCVA) of 0.7/1 in both eyes and intraocular pressure (IOP) of less than 16 mmHg in both eyes. Slit-lamp examination revealed a suspicious conjunctival lesion in the OD, initially suspected of being a lymphoma (**Fig. 1A, B**).

A conjunctival biopsy was performed, revealing inflammatory changes without evidence of malignancy. The patient was scheduled for surgical excision and placement of an amniotic membrane graft at the biopsy site.

Two months later, during follow-up, the patient showed bilateral facial hemispasm, severe headaches, and orbital discomfort. The case was discussed with neurology, and an orbital magnetic resonance (MRI) was performed, which ruled out orbital infiltration or involvement of the extrinsic ocular muscles and globe structures. Only conjunctival thickening with hyperenhancement was noted. Lacrimal glands and ducts were normal.

A month later, the patient returned to the emergency department with increased discomfort and decreased visual acuity. VA in OD was reduced to counting fingers, with only conjunctival hyperemia observed on slit-lamp examination and a 1.5 mm hyphema noted in the anterior chamber. OD IOP remained normal. Topical corticosteroids were prescribed with a tapering schedule, along with cycloplegic drops, and acenocoumarol was discontinued.

One week later, the hyphema resolved, and VA improved to 0.6/1 in OD and 1/1 in OS.

Neurological evaluation for facial spasms and headache was normal. A follow-up MRI ruled out central nervous involvement, showing only the known ocular infiltration.

The excision of the affected conjunctival area was performed with an amniotic membrane graft applied to the excised area.

The pathology report was positive for moderately differentiated invasive squamous cell carcinoma. No lymphovascular invasion was observed, although the lesion occupied the entire sample submitted.

Clinically, the patient’s pain increased, which made it increasingly difficult for him to cooperate during the examination. Endothelial precipitates were observed on slit-lamp examination, but no new changes were noted.

Treatment with interferon was proposed, but topical mitomycin treatment was ultimately prescribed due to a shortage of interferon.

One week later, a cyclitic membrane was observed on slit-lamp examination, and the patient was diagnosed with uveitis. Topical corticosteroids and cycloplegic eye drops were prescribed.

The patient’s pain continued to increase, so the oncology department was contacted to adjust pain management. A few days later, the patient showed clinical improvement in both uveitis and pain. Vision in the right eye at this point was counting fingers at one meter, while vision in the left eye remained stable. IOP also remained stable.

A few days later, inferior corneal edema and neovascularization were observed. Another MRI was performed, showing significant signal alteration with contrast enhancement in the anterior aspect of the right eyeball, with probable focal extension to the eyelid and likely infiltration of the right lacrimal gland. Infiltration of the extrinsic muscle insertions of the right eye was also noted, along with infiltration of the eyeball wall with posteroinferior extension. The optic nerve was not involved. Additionally, a probable retinal detachment was described, approximately 5 mm in maximum thickness in the inferolateral quadrant, with signal alterations corresponding to the vitreous and aqueous humor of the right eye. The right orbit and ocular structures were otherwise normal.

Given the MRI results, the case was presented to the tumor board, which decided on a total orbital exenteration, which was performed a few days later. Orbital reconstruction was not performed until histopathological evaluation of the extent of the lesion was completed.

**Fig. 2:**
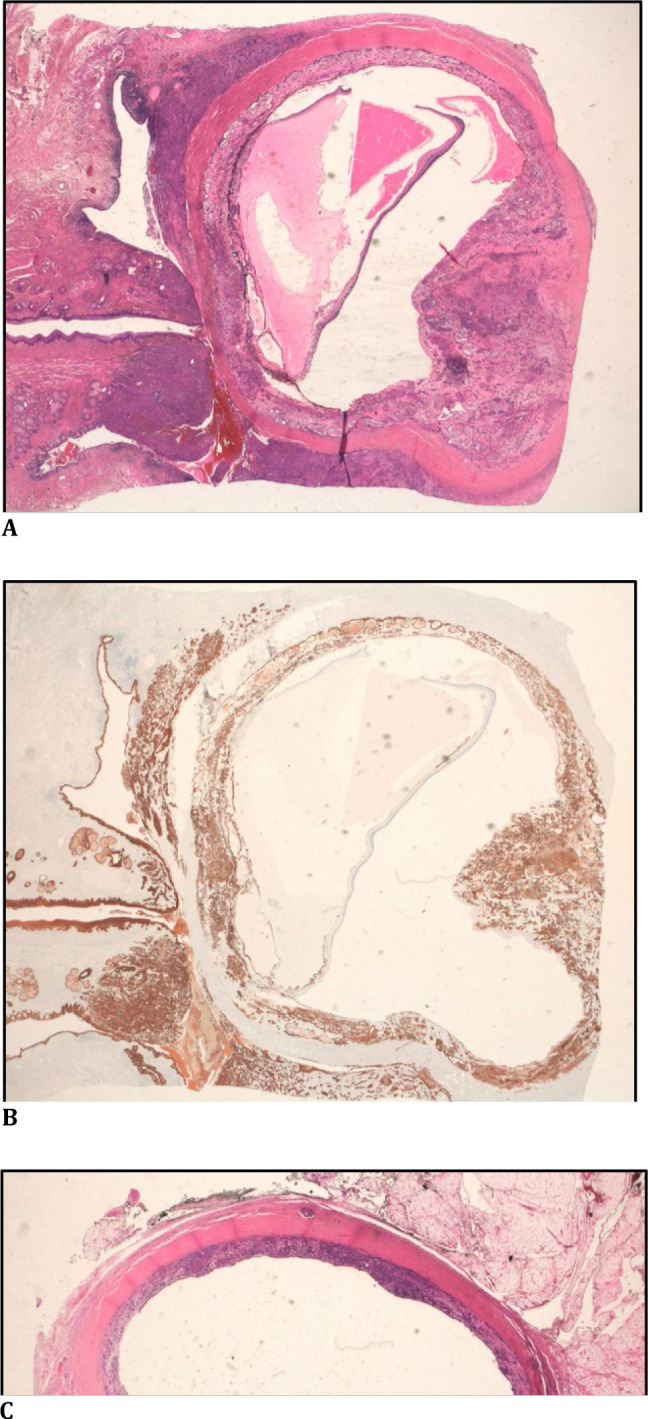
**A-B**. Section of the eyeball and eyelids showing the neoplasm at the conjunctival level (*) and its extension to the entire wall of the eyeball (A: HE, 10X / B: P63, 10X); **C**. Detail of the eyeball wall with the extension of the neoplasm at the retroretinal level (HE, 40X)

Histopathological examination of the exenteration specimen revealed an infiltrative neoplasm with poorly differentiated squamous areas, associated with regions showing a tubuloglandular pattern. The neoplasm extensively affected the bulbar and tarsal conjunctiva, the lacrimal gland, as well as the fibroadipose structures of the eyelids, primarily involving superficial structures. Furthermore, the neoplasm extended into the choroid, affecting nearly the entire choroidal layer (**Fig. 2A-C**).

The surgical margins and the optic nerve were free from involvement.

**Fig. 3:**
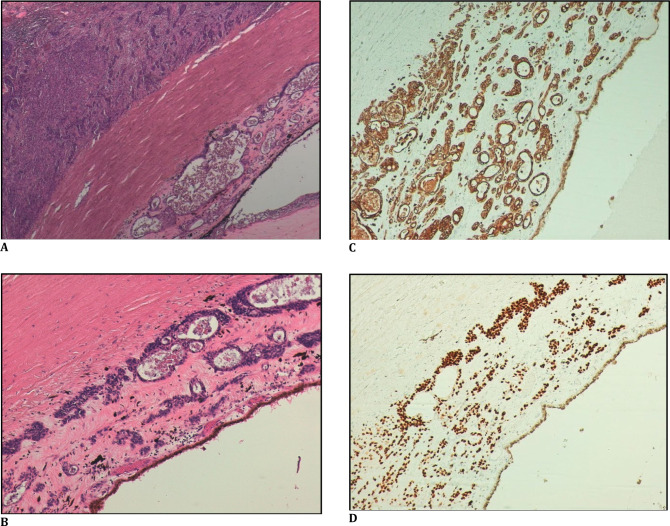
**A**. Section showing the squamous component external to the sclera (*) and the tubuloglandular component internal to the sclera (#); **B-D**. Detail of the tubuloglandular pattern with circumferential retroretinal extension (B: HE, 100X / C: CKAE1-AE3, 100X / D: P63, 100X)

In the immunohistochemical study, both components exhibited diffuse positivity for CKAE1-AE3 and p63 (**Fig. 2A-C, 3A-D**), with patchy expression of CK7, BEREP4, and synaptophysin, and a Ki-67 proliferative index of approximately 40%.

The neoplasm was ultimately classified as adenosquamous carcinoma (mucoepidermoid carcinoma) of the bulbar-palpebral conjunctiva, with both components being moderately differentiated (**Fig. 3A-D**).

**Fig. 4:**
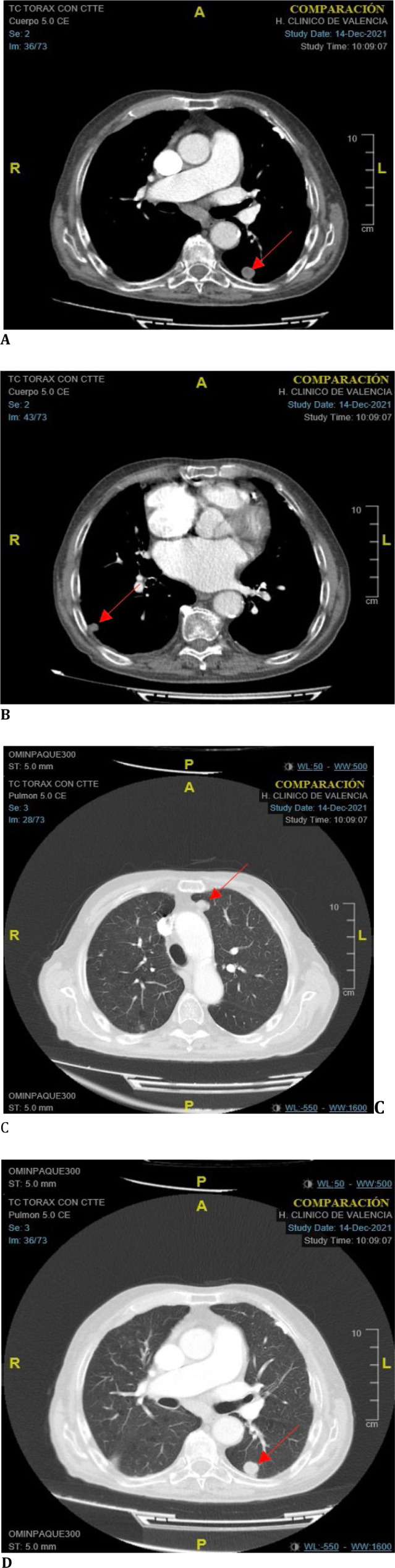
**A-D** Chest CT revealing multiple bilateral pulmonary nodules and mediastinal lymphadenopathy suggestive of metastatic disease

A complete CT scan was performed, revealing newly developed bilateral pulmonary nodules compatible with metastases, along with significant subcarinal and hilar adenopathies (**Fig. 4A-D**).

A PET-CT scan was performed, which also revealed multiple bilateral hypermetabolic pulmonary nodules, highlighting three large ones in the right lower lobe, the superior segment of the left lower lobe, and the anterior region of the left upper lobe, seated in the paramediastinal pleura. A hypermetabolic pleuropulmonary focus was also described in the anterobasal region of the lingula, along with a hypermetabolic endobronchial lesion in the right upper lobar bronchus. These findings were consistent with the previously described CT scan.

In the abdomen, the PET-CT revealed extensive hypermetabolic tumor involvement in the liver parenchyma, especially in the left lobe and segment 4. Additionally, hepatic foci were identified in segments 5, 7, and 8, along with active adenopathy in the hepatic hilum and portocaval region. A small metastatic bone focus was also described in the D5 vertebra.

At this point, the patient reported significant subjective pain relief following the surgery.

The pulmonology department performed bronchial aspiration cytology (BAC), which was negative for malignancy. However, an analysis of subcarinal adenopathy (area 7) revealed infiltration by non-small cell carcinoma, compatible with metastasis from the primary conjunctival carcinoma.

The oncology department concluded that the patient was experiencing a generalized oncological process. The patient passed away two months later.

## Discussion

Squamous cell carcinoma of the conjunctiva is a tumor that can be locally aggressive in the orbit, especially with late diagnosis [**11**], but rarely produces regional or distant orbital extension if treated early and appropriately with surgery, adjuvant techniques, and proper histopathological analysis of the lesion [**1,2,6,11**].

The risk of recurrence and metastasis is also associated with two specific histopathological variants: sarcomatous or spindle cell carcinoma, for which one case of lung metastasis has been reported, and the mucoepidermoid variant, for which we also found an isolated case of lung metastasis [**10**]. The latter is also known for its greater local aggressiveness in the orbit, higher recurrence rate after local excision of the initial lesion, and a higher number of locoregional lymph node metastases [**2,7,8,10,11**].

Although distant metastasis in these subtypes remains extremely rare, increased vigilance for clinically suspicious lesions, along with early intervention and histopathological analysis, could lead to more aggressive initial treatments, thereby potentially improving the long-term prognosis and quality of life for certain patients.

## Conclusions

We presented a case of mucoepidermoid carcinoma of the conjunctiva with an unusual circumferential choroidal infiltrative pattern and aggressive evolution with pulmonary metastasis. It is essential to screen for suspicious conjunctival lesions by performing deep biopsies to reduce sampling bias in large lesions and to avoid underdiagnosis of uncommon aggressive mixed histological subtypes, such as the described mucoepidermoid carcinoma, which, in rare instances, could lead to fatal outcomes. Additionally, imaging tests at the thoracoabdominal level should be conducted to screen for metastatic lesions, especially when dealing with more aggressive variants.
